# Maternal Consumption of Hesperidin and Naringin Flavanones Exerts Transient Effects to Tibia Bone Structure in Female CD-1 Offspring

**DOI:** 10.3390/nu9030250

**Published:** 2017-03-08

**Authors:** Sandra M. Sacco, Caitlin Saint, Paul J. LeBlanc, Wendy E. Ward

**Affiliations:** 1 Department of Kinesiology, Faculty of Applied Health Sciences, Brock University, St. Catharines, ON L2S 3A1, Canada; ssacco@brocku.ca; 2 Centre for Bone and Muscle Health, Brock University, St. Catharines, ON L2S 3A1, Canada; caitlinsaint@trentu.ca (C.S.); pleblanc@brocku.ca (P.J.L.); 3 Department of Health Sciences, Faculty of Applied Health Sciences, Brock University, St. Catharines, ON L2S 3A1, Canada

**Keywords:** bone development, flavanones, hesperidin, mice, naringin, structure

## Abstract

Hesperidin (HSP) and naringin (NAR), flavanones rich in citrus fruits, support skeletal integrity in adult and aging rodent models. This study determined whether maternal consumption of HSP and NAR favorably programs bone development, resulting in higher bone mineral density (BMD) and greater structure and biomechanical strength (i.e., peak load) in female offspring. Female CD-1 mice were fed a control diet or a HSP + NAR diet five weeks before pregnancy and throughout pregnancy and lactation. At weaning, female offspring were fed a control diet until six months of age. The structure and BMD of the proximal tibia were measured longitudinally using in vivo micro-computed tomography at 2, 4, and 6 months of age. The trabecular bone structure at two and four months and the trabecular BMD at four months were compromised at the proximal tibia in mice exposed to HSP and NAR compared to the control diet (*p <* 0.001). At six months of age, these differences in trabecular structure and BMD at the proximal tibia had disappeared. At 6 months of age, the tibia midpoint peak load, BMD, structure, and the peak load of lumbar vertebrae and femurs were similar (*p >* 0.05) between the HSP + NAR and control groups. In conclusion, maternal consumption of HSP and NAR does not enhance bone development in female CD-1 offspring.

## 1. Introduction

Bioactives, naturally present in foods, may provide a dietary strategy to support healthy bone development. In a developing mouse model, exposure to soy isoflavones during early postnatal life, but not maternal exposure, sets a trajectory for higher bone mineral density (BMD), improved bone structure, and greater bone strength in female offspring at adulthood [1,2,3,4]. Other bioactives such as citrus flavanones (i.e., hesperidin (HSP) and naringin (NAR)) have been shown to exert bone-sparing effects in adult and aging rodents [5,6,7,8,9,10,11], but whether maternal consumption of citrus flavanones during pregnancy and lactation changes the trajectory of bone development of offspring, resulting in stronger, healthier bones at adulthood has not been investigated. Given that the metabolites of citrus flavanones (i.e., hesperetin and naringenin) can be transferred to human offspring via mother’s milk [12], it is of interest to examine whether maternal exposure to HSP and NAR programs the bone tissue in adult female mouse offspring. 

HSP (hesperetin-7-rutinoside) and NAR (naringenin-7-neohesperidoside) are flavanone glycosides found in high amounts in citrus fruits. HSP is found in particularly rich amounts in oranges while NAR is present at high levels in grapefruits [13,14,15]. When consumed by humans and rodents, HSP and NAR are hydrolyzed into the aglycones, hesperetin and naringenin, and metabolized mainly into glucuronide forms by intestinal and liver conjugation [9,13,14,16,17]. The conjugated and aglycone metabolites circulate the body and modulate bone cell activity; in rat primary osteoblasts, hesperetin and hesperitin-7-*O*-glucuronide, at levels that are physiologically achievable through dietary consumption, increased osteoblast differentiation [18,19], and increased the mRNA expression of bone morphogenic proteins (BMPs) [18,19], runt-related transcription factor 2 (Runx2) [19], and osterix [18,19] that promote osteoblast differentiation. In murine MC3T3-E1 osteoblast cells [20] and rat [10] and human [21] bone mesenchymal stem cells, NAR increased osteoblast differentiation and proliferation and increased the expression of BMP-2 [20], osteopontin [21], and osteocalcin [10]. No data exists on the ability for HSP, NAR, or their metabolites to epigenetically regulate the transcriptions of genes involved in bone development. However, that HSP and NAR stimulate signaling pathways implicated in stimulating bone formation provides biological plausibility for HSP and NAR to support bone development and skeletal integrity. 

The majority of our knowledge on the positive effects of HSP and NAR on bone is derived from rodent models of bone loss (i.e., ovariectomy, orchidectomy) [5,6,7,8,10,11] and aging (i.e., senescence) [9]. While intake of HSP and NAR is reported to benefit bone health in male and female rodents, most research has been performed in females. These studies demonstrate that dietary HSP and NAR protect against the loss of bone mineral and the deterioration of bone structure and strength during ovariectomy [6,7,8,10,11]. However, one study [7] in younger (3 months old) intact female rats reported gains in femoral BMD, which agrees with other work demonstrating that HSP supports bone cell function via modulating signaling proteins and bone cell differentiation to increase bone formation [18]. However, no studies have assessed whether maternal consumption of HSP and NAR affects bone development in growing and adult offspring. Given that treatment with HSP, NAR, or their combination stimulates bone formation [10,18], supports bone mineral accrual [7], and protects against bone loss in ovariectomized [6,7,8,10,11] female rodents, we hypothesized that maternal consumption of HSP and NAR before and during pregnancy and throughout lactation would set a trajectory for better bone health in female offspring. Thus, the objective of the present study was to determine whether maternal consumption of HSP and NAR results in higher BMD, improved bone structure, and greater bone strength in female CD-1 offspring at adulthood.

## 2. Materials and Methods

### 2.1. Animals and Diets

The experimental protocol (AUP 14-04-01, 2014) was approved by the Animal Care Committee at Brock University, St. Catharines, ON, Canada. Five-week-old female (*n =* 17) and 8-week-old male (*n =* 8) CD-1 mice were purchased from Charles River Canada (St. Constant, QC, Canada) and housed with 4–5 mice per cage at 22 °C–24 °C, 50% humidity with a 12:12-h light:dark cycle. Female mice were randomized to the American Institute of Nutrition-93 (AIN-93G) control diet (CON, *n =* 9) for growth, pregnancy and lactation [22] or the CON diet supplemented with 0.5% HSP + 0.25% NAR (HSP + NAR, *n =* 8). These doses reflect a moderate (400 mL) to high (1 L) daily intake of orange or grapefruit juices in humans [14,17] and represent doses that have been shown to exert protection to bone health in adult and aging rodents [5,6,7,8,9]. The AIN-93G diet used in the present study was modified to contain alcohol-extracted casein (TD. 06706, Harlan Teklad, Mississauga, ON, Canada) to remove vitamins that are naturally-occurring in casein and that may influence bone development. HSP (H5254, Sigma Aldrich, Oakville, ON, Canada) and NAR (N1376, Sigma Aldrich, Oakville, ON, Canada) were added to the CON diet at the expense of cornstarch. All mice had free access to food and water throughout the study, and food intake was measured twice per week using an electronic scale.

### 2.2. Study Design

At 10 weeks of age, mice were mated harem-style by housing 2–3 females with one male. Mating pairs were maintained on the female’s respective diets, and, once identified as pregnant, female mice were housed individually and remained on their respective diets during the remainder of pregnancy and throughout lactation. Offspring were weighed at post-natal day (PND) 9, PND 16, and PND 21 using an electronic scale. At weaning, female offspring were housed with five mice per cage and switched to the CON diet for the remainder of the study duration. Food intake was measured twice per week and body weights measured weekly to monitor growth.

### 2.3. In Vivo Analysis of Structure of Tibias by μCT

At 2, 4, and 6 months of age, mouse offspring were anesthetized using 2%–5% isoflurane dissolved in 100% oxygen, and the right tibias were scanned in vivo using micro-computed tomography scanning (μCT) (Skyscan 1176, Bruker microCT, Kontich, Belgium) and host software (1176 version 1.1, Skyscan 1176, Bruker microCT, Kontich, Belgium), as previously described [23]. Scanning of the right proximal tibia was performed using an isotropic voxel size of 9 μm^3^, 1 mm aluminum filter, 40 kV tube voltage, 300 μA amperage, a rotation step of 0.8°, 3350 ms integration time, and over 180° with no frame averaging. Each scan lasted 16 min 23 s and resulted in 460 mGy exposure per scan to the scanned tibias (TN-502RD-H, Best Medical Canada, Ottawa, ON, Canada). This dose of radiation does not affect tibial bone structure when tibias are repeatedly exposed at 2, 4, and 6 months of age [23]. At 6 months of age, animals were euthanized within five days after receiving their last scan by exsanguination under anesthesia (5% isoflurane dissolved in 100% oxygen), followed by cervical dislocation. Tibias, femurs, and lumbar vertebrae (LV 1–6) were excised, cleaned of soft tissue, and then wrapped in saline soaked gauze and stored at −80 °C for future analyses. 

### 2.4. Ex Vivo Analysis of Structure of Femurs and Lumbar Vertebrae by μCT

Right femurs and the second lumber vertebra (LV2) were scanned ex vivo using μCT scanning (Skyscan 1176, Bruker microCT, Kontich, Belgium). These sites were scanned ex vivo to minimize the level of radiation exposure to each mouse. The bones were wrapped in parafilm to retain moisture during scanning and placed axially in a foam holder. The foam holder containing the bones was then secured to a mouse bed for ex vivo scanning using a 9 µm^3^ isotropic voxel size, 0.25 mm aluminum filter, voltage of 45 kVp, tube current of 545 µA, 850 ms exposure time, and 0.2° rotation step. Scans were performed over 180° without using frame averaging.

### 2.5. Reconstruction of In Vivo and Ex Vivo Images Obtained Using μCT

Graphics Processing Unit (GPU)-acceleration (GPUReconServer, Skyscan, Bruker microCT, Kontich, Belgium) and NRecon Reconstruction 64-bit software (Skyscan, Bruker microCT, Kontich, Belgium) were used to reconstruct scanned images. Except for variable misalignment compensations, all scanned samples within each skeletal site received the same corrections to smoothing, ring artifacts, beam hardening, and defect pixel masking. Reconstructed images were then reoriented (DataViewer software, version 1.5.0, Skyscan, Bruker microCT, Kontich, Belgium), and the transaxial images were saved. 

### 2.6. Regions of Interest (ROI) and Segmentation of In Vivo and Ex Vivo Images Obtained Using μCT

ROIs were selected from transaxial images and saved as new datasets using CTAnalyzer software (Skyscan Bruker microCT, Kontich, Belgium). At the proximal tibia, the ROI consisted of the trabecular and cortical bone and began 0.967 mm (110 slices) away from the metaphyseal side of the growth plate and extended 0.510 mm (58 transaxial slices) towards the ankle [23]. At the femur neck, an ROI of the trabecular region was manually drawn to a few pixels away from the endocortical boundary and consisted of 1.680 mm (191 slices), starting at the distal edge of the femur head and extending towards the diaphysis of the femur. At the distal femur, the ROI of the trabecular region was manually drawn a few pixels away from the endocortical boundary and consisted of 0.879 mm (100 slices), starting 0.616 (70 slices) proximal to the metaphyseal edge of the growth plate and extending toward the femur neck. Another ROI was manually drawn around the midpoint region of the femur, rich in cortical bone, and consisted of 0.879 mm (100 slices) spanning above and below the femur midpoint. At the LV2, ROIs of the trabecular and cortical regions of the vertebral bodies were manually and consisted of 115 transverse slices (1.011 mm), spanning above and below the midpoint of LV2. 

Task lists were generated and applied (CTAnalyzer software, Skyscan Bruker microCT, Kontich, Belgium) to the saved datasets to segment bone from the background and to analyze trabecular or cortical bone structure at each skeletal site. At the proximal tibia, an automated task list was generated [23] to segment the trabecular bone from the cortical bone. Trabecular and cortical bone were then segmented as previously described [23]. At the femur neck, femur midpoint, distal femur, and LV, task lists were generated to segment the trabecular and cortical bones from the background. Local thresholding was used to segment the trabecular bone from the background (femur neck: lower threshold = 74, upper threshold = 255, radius = 6, constant = 0; distal femur: lower threshold = 71, upper threshold = 255, radius = 6, constant = 0; LV: lower threshold = 80, upper threshold = 255, radius = 7, constant = 0) and global thresholding (femur: lower threshold = 124, upper threshold = 255; LV2: lower threshold = 122, upper threshold = 255) for segmenting the cortical bone from the background at the femur midpoint and LV2 [24].

The trabecular bone outcome measures determined at the proximal tibia, femur neck, distal femur, and LV included bone volume (BV, mm^3^), total volume (TV, mm^3^), bone volume fraction (BV/TV, %), trabecular thickness (Tb.Th, mm), trabecular number (Tb.N, mm-1), trabecular separation (Tb.Sp, mm), degree of anisotropy (DA, no unit), and connectivity density (Conn.D, 1/mm^3^). Cortical bone outcome measures at the tibia, femur midpoint and LV included total cross-sectional area inside the periosteal envelope (Tt.Ar, mm^2^), cortical bone area (Ct.Ar, mm^2^), cortical area fraction (Ct.Ar/Tt.Ar, %), average cortical thickness (Ct.Th, mm), periosteal perimeter (Ps.Pm, mm), endocortical perimeter (Ec.Pm, mm), marrow area (Ma.Ar, mm^2^), and eccentricity (no unit). 

### 2.7. Bone Mineral Density (BMD) and Peak Load of Tibias, Femurs and LV1-3 

To determine the trabecular BMD and cortical tissue mineral density (TMD) at the proximal tibia that were scanned in vivo at 2, 4, and 6 months of age, calibration phantoms consisting of 0.25 and 0.75 g/cm^3^ calcium hydroxyapatite were scanned and reconstructed using the exact same parameters as all in vivo scans. BMD was then calibrated against the attenuation coefficient, and the trabecular BMD and cortical TMD at the proximal tibia were then measured against the attenuation coefficient [25]. Since measurement of BMD using µCT scanning is often not possible in excised samples due to the introduction of air bubbles from storage [25], BMD of the right femurs and LV1-3 was measured by dual energy X-ray absorptiometry (DXA) (Orthometrix, White Plains, NY, USA) and a specialized software program (Host Software version 3.9.4; Scanner Software version 1.2.0, Orthometrix, White Plains, NY, USA). Calibration of the DXA was performed daily using a calibration phantom. Femurs and LV1-3 were scanned using a resolution of 0.01 mm × 0.01 mm and at a speed of 2 mm/s. To determine bone strength at skeletal sites rich in cortical bone, the peak load at the midpoint of the right tibia and right femur was determined by 3-point bending using a Materials Testing System (Model 4442, Instron Corp., Norwood, MA, USA) and specialized software (Bluehill 2, Instron Corp., Norwood, MA, USA) [26]. The peak load at skeletal sites rich in trabecular bone was determined by compression testing at the femur neck and LV2 [26]. 

### 2.8. Statistical Analyses

All statistical analyses were performed using SPSS Statistics (version 22, IBM, Armonk, NY, USA). The effects of diet, age, and their interaction on food intake, body weight, and in vivo trabecular and cortical bone structure were determined using a repeated measures analysis of variance (ANOVA) (general linear model). Comparisons on all bone outcome measures between CON and HSP + NAR groups within each time point were assessed by unpaired two-tailed *t*-tests. A repeated measures ANOVA (general linear model) with a Bonferroni post-hoc test was used to determine longitudinal changes within the trabecular and cortical bone structural outcomes within each group. The primary outcome included BV/TV at the proximal tibia, with a total of seven mice per treatment group required to achieve a power of 0.8 and an alpha of 0.5. Missing values that resulted from mouse leg movement during the scan or due to computer error were replaced with the series mean. This occurred a total of four times (once in the CON group at 2 months of age, twice in the CON group at 4 months of age, and once in the HSP + NAR group at 4 months of age). Results are expressed as mean ± standard error of the mean (SEM) and the significance level was set at *p* < 0.05.

## 3. Results

### 3.1. Food Intake and Body Weight

Litter sizes (CON = 14 ± 2 pups/L, HSP + NAR= 13 ± 2 pups/L) were similar between the CON and HSP + NAR groups (p > 0.05). In addition, no differences (p > 0.05) in litter weight at PND9 (CON = 83 ± 10 g, HSP + NAR = 82 ± 6 g), PND16 (CON = 118 ± 10 g, HSP + NAR = 118 ± 5 g), or at weaning (PND21, CON = 162 ± 17 g, HSP + NAR = 160 ± 13 g) were observed between the groups. These results demonstrate that maternal consumption of HSP + NAR does not affect the growth patterns of female CD-1 offspring during suckling. 

**Figure 1:**
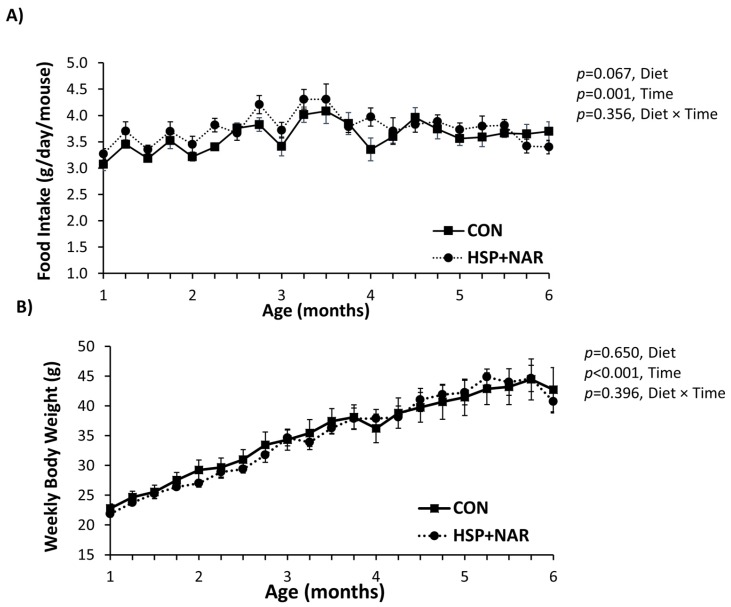
Mean daily food intake (**A**) and body weight (**B**) of female mice whose mothers were fed a control diet (CON) or a diet consisting of a combination of 0.5% hesperidin (HSP) and 0.25% naringin (NAR) before and during pregnancy and lactation, *n =* 8–9 per group. Mean ± standard error of the mean (SEM).

After weaning, an effect of time was observed for the mean daily food intake (*p <* 0.01) (Figure 1A) and weekly body weight (*p <* 0.001) (Figure 1B), which was expected because the mice were growing. There was no diet or time x diet effect on daily food intake or body weight throughout the study (*p >* 0.05). At the end of the study, the final body weights in 6-month-old offspring did not differ between CON and HSP + NAR groups. Thus, the food intake or growth characteristics of female offspring from weaning until six months of age were not affected by maternal and suckling exposure to HSP + NAR.

### 3.2. In Vivo Measurements of Trabecular and Cortical Bone Mineral Density (BMD) and Bone Structure at the Proximal Tibia 

**Figure 2:**
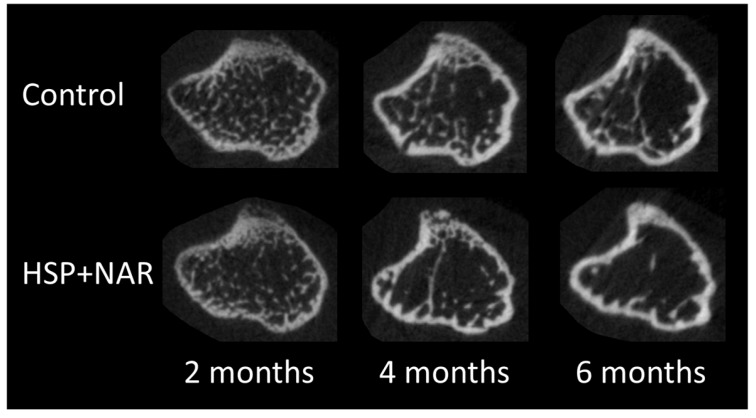
Representative grayscale transaxial images of right proximal tibias of female CD-1 mouse offspring at 2, 4, and 6 months of age.

At the proximal tibia, a significant effect of diet (*p <* 0.05) was observed for several trabecular structural outcomes, whereby HSP + NAR resulted in lower BV, BV/TV, Tb.N, and Conn.D and higher Tb.Sp compared to CON at both 2 and 4 months of age (Table 1, Figure 2). A significant interaction between diet and time (*p <* 0.05) was observed for BMD and Tb.Sp, whereby HSP + NAR resulted in lower BMD at 4 months of age and higher Tb.Sp at 2 and 4 months of age compared to the CON group. No effects of diet on the trabecular bone structure at the proximal tibia was observed at 6 months of age. Thus, maternal consumption of HSP + NAR during pregnancy and lactation compromises trabecular BMD and bone structure at the proximal tibia in developing female CD-1 offspring; however, this effect does not persist into adulthood. In addition to an effect of diet, an effect of time (*p <* 0.001) was observed, whereby offspring from both the CON and HSP + NAR groups experienced a decrease in TV, BV, BV/TV, Tb.N, and Conn.D and an increase in Tb.Th and Tb.Sp from 2 to 4 months of age (Table 1, Figure 2). Similar to these changes, trabecular BMD from HSP + NAR exposure decreased from 2 to 4 months of age, while BMD decreased in the CON group after 4 months of age (*p <* 0.001). Diminutions in BV, BV/TV, Tb.N, and Conn.D within the CON group persisted to 6 months of age, resulting in trabecular bone structural outcomes that were significantly lower compared to both the 2- and 4-month time points (*p <* 0.05). In the HSP + NAR group, changes in trabecular BMD and bone structure were maintained from 4 to 6 months of age. Thus, female CD-1 offspring achieve peak trabecular BMD and bone structure before 4 months of age, a finding that agrees with previous work [23].

With regards to the cortical bone structure at the proximal tibia, Ct.Ar was significantly lower (*p <* 0.05) in the HSP + NAR group compared to the CON group at 4 months of age (Table 1, Figure 2). In addition, a time x diet effect was observed (*p <* 0.05) for Ps.Pm at the proximal tibia, whereby HSP + NAR resulted in lower Ps.Pm at 2 months of age compared to CON (*p <* 0.05). However, this effect was not apparent at 4 and 6 months of age. No effects (*p >* 0.05) from HSP + NAR exposure on cortical TMD were observed. Thus, maternal exposure during pregnancy and lactation to a combined 0.5% HSP and 0.25% NAR diet delays some characteristics of cortical bone development in female CD-1 offspring. In addition to an effect of diet on cortical bone structure, an effect of time (*p <* 0.001) was observed, whereby augments to the cortical bone TMD and structure, including Ct.Ar, Ct.Ar/Tt.Ar, and Ct.Th, were observed from 2 to 6 months of age in the CON and HSP + NAR groups (Table 1, Figure 2). In addition, diminution of Tt.Ar, Ec.Pm, and Ma.Ar were observed in both groups. These findings agree with our previous work showing that cortical bone structure reaches its peak at a later time point compared to trabecular bone in female CD-1 mice [23].

### 3.3. Ex Vivo Measurements of Trabecular and Cortical Bone Structure

**Figure 3:**
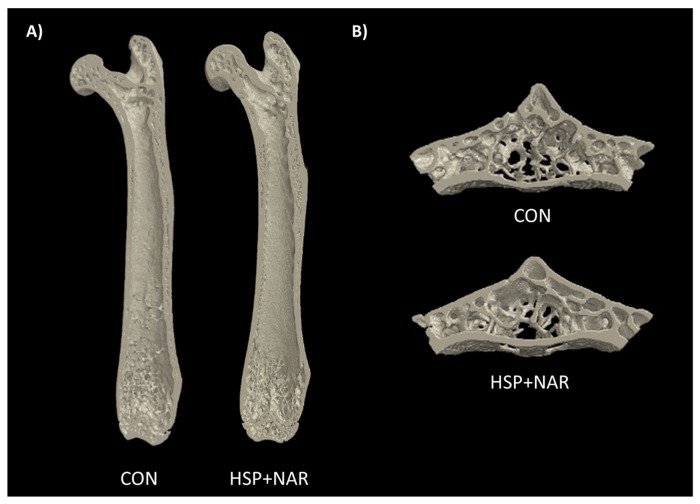
Representative images of the right femur (**A**) and the second lumbar vertebra (LV2) (**B**) of 6-month-old female mice whose mothers were exposed to control (CON) or 0.5% hesperidin (HSP) and 0.25% naringin (NAR) diets through pregnancy and lactation. Coronal sections consisting of the ventral half of the femur are depicted in (**A**); Transverse sections consisting of 1.011 mm of the vertebral body midsection are depicted in (**B**).

At the femur neck, there were no significant differences between the CON and HSP + NAR groups for BV/TV, Tb.Th, Tb.N, and Tb.Sp (Table 2, Figure 3A). Other trabecular parameters including BV, TV, Conn.D, and DA did not differ between groups. In addition, no differences in trabecular bone properties (*p >* 0.05) were observed at the distal metaphyseal region of the femur (Table 2). At the femur midpoint, there were no differences in cortical bone properties including Ct.Ar and Ct.Th (Table 2, Figure 3A), as well as Tt.Ar, Ct.Ar/Tt.Ar, Ps.Pm, Ec.Pm, Ma.Ar, or Ecc between the CON and HSP + NAR groups.

At LV2, there were no significant differences between CON and HSP + NAR with regards to BV/TV, Tb.Th, Tb.N, Tb.Sp (Table 3, Figure 3B), or other trabecular parameters. Similar to the trabecular bone, the cortical bone structural properties at LV2, including Ct.Ar and Ct.Th, were similar (*p <* 0.05) between the CON and HSP + NAR groups (Table 3). No other differences in cortical bone structure were observed between the groups. Collectively, these data indicate that maternal exposure to HSP and NAR does not result in long-term consequences to the trabecular or cortical bone structure at multiple skeletal sites in female CD-1 offspring. 

### 3.4. Ex Vivo Measurement of Bone Mineral Density (BMD) and Peak Load

No significant differences (*p >* 0.05) in BMD at the whole femur (Table 2) or LV1-3 (Table 3) were observed between the CON and HSP + NAR groups at six months of age. The peak load at the tibia midpoint (CO*N =* 18.1 ± 1.3 N, HSP + NAR= 18.1 ± 1.2) and at the femur midpoint, femur neck, and LV2 did not differ (*p >* 0.05) between the CON and HSP + NAR groups (Table 2 and Table 3). 

## 4. Discussion

Our data demonstrates that maternal consumption of HSP and NAR compromises trabecular structure at the proximal tibia in female CD-1 offspring at two and four months of age and trabecular BMD at four months of age; however, these differences do not persist to six months of age. At the femur and LV, the trabecular and cortical BMD and structure were not affected by dietary HSP and NAR. Moreover, the peak load was not altered by dietary HSP and NAR at the three skeletal sites measured (i.e. tibia midpoint, femur midpoint, and LV2). Thus, maternal consumption of HSP and NAR during pregnancy and lactation exerts transient effects on trabecular BMD and bone structure in female CD-1 offspring post-weaning, but these effects do not result in long-term detriments to skeletal integrity and strength at adulthood. 

A novel aspect of this study included the timing of exposure to HSP and NAR. Previous studies have not investigated the effect of maternal exposure to these citrus flavanones on offspring. Thus, findings from the present study suggest that there is a critical period before and/or during pregnancy and/or lactation that transiently compromises the trabecular bone structure in female offspring. Determining which of these period(s) are critical for the effects of HSP and/or NAR on skeletal development is an area of future research interest. In addition, our findings suggest that HSP + NAR exerts differential effects on bone tissue that is dependent on the timing of exposure, as others have demonstrated that HSP, NAR, or their aglycones support bone health in mouse and rat models of adulthood, aging, or surgically- or senescent-induced bone loss [5,6,7,8,9,10,11]. For example, in 3-month-old female rats, a daily intake of 0.5% HSP increased femoral BMD by six months of age [7]. In ovariectomized mouse [8,11] and rat [6,7,10] models of postmenopausal bone loss, HSP, NAR or their combination protected against the deterioration of BMD, bone structure, and biomechanical strength. The protective effects of HSP and NAR on the trabecular bone have also been observed in orchidectomized mice [5] and senescent rats [9]. Collectively, these studies demonstrate that HSP, NAR, or their combination support the accrual and maintenance of BMD, bone structure, and strength in both adult and aging rodents at doses [5,6,7,8,9] similar to those used in the present study. 

The mechanism of action is currently unknown. Studies have not investigated the effects of HSP, NAR, or their metabolites on their potential to regulate the transcription of genes involved in bone development through epigenetic mechanisms. The action of HSP and NAR on bone cell function is largely positive and attributed to their aglycone or glucuronide forms acting to increase the mRNA expression of BMP-2, BMP-4, Runx2, and osterix, which are involved in driving osteoblast differentiation and facilitating bone formation [18,19,20]. HSP and NAR may also decrease osteoclast activity and bone resorption [9,11]. While some literature has indicated HSP and NAR metabolites as exhibiting estrogenic properties [11,27], others have demonstrated that they do not activate estrogen receptor-alpha- (ER-alpha) or ER-beta-mediated transcription of genes [11] and that their binding affinities to estrogen receptors are low [28]. Thus, the estrogenic or anti-estrogenic potential of HSP and NAR is uncertain. Moreover, whether HSP and/or NAR metabolites can cross the placenta or be transferred to the offspring via the mother’s milk in CD-1 mice is an area of future investigation. It is possible that maternal exposure to HSP and/or NAR modulates circulating levels of calciotropic hormones in the mother to result in alterations to calcium availability to the fetus in utero or to offspring during suckling; however, this remains to be determined. Further investigation is necessary to determine the mechanisms whereby HSP + NAR compromises trabecular bone structure at the proximal tibia in two- and four-month-old female offspring. Moreover, further investigation is warranted to determine whether the skeletal tissue of male offspring also responds to maternal exposure to HSP + NAR.

Among the strengths of the present study is the comprehensive assessment that was performed on BMD, structure, and strength at multiple skeletal sites. That HSP + NAR exposure did not induce effects at 6 months of age on bone mineral, structure, and biomechanical strength at the tibias, femurs, and LV demonstrates consistency among all skeletal sites examined and provides strong evidence that HSP + NAR does not have long-lasting effects on skeletal integrity in female CD-1 offspring when mothers are exposed before and during pregnancy and throughout lactation. Another strength is that that changes in tibial BMD and bone structure were evaluated within the same mice throughout the course of the study. This longitudinal evaluation of BMD and bone structure within the same mice resulted in a statistically powerful design and reduced the number of animals required to conduct the present study. A limitation of the present study is the uncertainty of whether the compromised bone structure with HSP + NAR treatment at two and four months of age is due to the combined action of HSP and NAR or if one of these flavanones is responsible for these observed effects. As we are the first to examine the efficacy HSP and NAR in a programming model of bone development, we chose to provide HSP and NAR in combination at levels that have established effects in aging rodent models [8,9,11] as a proof of efficacy. Moreover, identifying whether the critical period is preconception, and/or during pregnancy, and/or lactation remains unknown. 

## 5. Conclusions

In conclusion, maternal consumption of HSP + NAR exerts transient effects on trabecular bone development at the proximal tibia in female CD-1 offspring. That BMD, structure, and strength at the tibia, femur, and LV were similar between the CON and HSP + NAR groups at six months of age suggests that maternal consumption of HSP + NAR does not favorably program bone health in female CD-1 offspring.

## Figures and Tables

**Table 1:** Bone mineral density (BMD) and structure at the proximal tibia at 2, 4, and 6 months of age in female CD-1 mice whose mothers were exposed to control (CON) or 0.5% hesperidin (HSP) and 0.25% naringin (NAR) diets through pregnancy and lactation.

Outcomes	CON			HSP + NAR			*p* Value		
	Age (Months)								
	2	4	6	2	4	6	Time	Diet	Time × Diet
**Trabecular Structure**									
BMD (g/cm^3^)	0.212 ± 0.007 ^a^	0.198 ± 0.003 ^a^	0.154 ± 0.006 ^b^	0.190 ± 0.010 ^a^	0.162 ± 0.008 ^†,b^	0.142 ± 0.008 ^b^	<0.001	0.007	0.015
TV (mm^3^)	1.185 ± 0.064 ^a^	0.936 ± 0.045 ^b^	0.898 ± 0.046 ^b^	1.022 ± 0.065 ^a^	0.853 ± 0.052 ^b^	0.831 ± 0.058 ^b^	<0.001	0.184	0.151
BV (mm^3^)	0.097 ± 0.010 ^a^	0.050 ± 0.006 ^b^	0.025 ± 0.004 ^c^	0.061 ± 0.009 ^†,a^	0.029 ± 0.005 ^†,b^	0.017 ± 0.004 ^b^	<0.001	0.013	0.099
BV/TV (%)	8.177 ± 0.645 ^a^	5.282 ± 0.464 ^b^	2.764 ± 0.298 ^c^	5.782 ± 0.698 ^†,a^	3.260 ± 0.434 ^†,b^	2.018 ± 0.526 ^b^	<0.001	0.011	0.161
Tb.Th (mm)	0.059 ± 0.001 ^b^	0.070 ± 0.001 ^a^	0.073 ± 0.001 ^a^	0.059 ± 0.001 ^b^	0.068 ± 0.002 ^a^	0.069 ± 0.002 ^a^	<0.001	0.243	0.406
Tb.N (1/mm)	1.386 ± 0.105 ^a^	0.751 ± 0.058 ^b^	0.379 ± 0.42 ^c^	0.976 ± 0.104 ^†,a^	0.479 ± 0.063 ^†,b^	0.284 ± 0.071 ^b^	<0.001	0.009	0.094
Tb.Sp (mm)	0.297 ± 0.010 ^c^	0.386 ± 0.003 ^b^	0.429 ± 0.006 ^a^	0.350 ± 0.015 ^†,b^	0.430 ± 0.008 ^†,a^	0.445 ± 0.006 ^a^	<0.001	0.002	0.004
DA (no unit)	2.334 ± 0.095 ^a^	1.970 ± 0.072 ^b^	2.150 ± 0.116 ^a,b^	2.562 ± 0.087	2.212 ± 0.126	2.962 ± 0.401	<0.001	0.030	0.305
Conn.D (1/mm^3^)	46.6 ± 4.7 ^a^	20.0 ± 2.1 ^b^	7.9 ± 1.8 ^c^	30.1 ± 2.1 ^†,a^	11.3 ± 3.1 ^†,b^	8.5 ± 2.3 ^b^	<0.001	0.004	0.051
**Cortical Structure**									
TMD (g/cm^3^)	0.802 ± 0.007 ^c^	0.941 ± 0.112 ^b^	0.996 ± 0.010 ^a^	0.810 ± 0.007 ^c^	0.932 ± 0.004 ^b^	0.995 ± 0.007 ^a^	<0.001	0.969	0.298
Ct.Ar (mm^2^)	1.295 ± 0.041 ^b^	1.396 ± 0.025 ^a,b^	1.456 ± 0.021 ^a^	1.230 ± 0.035 ^b^	1.299 ± 0.037 ^†,a^	1.367 ± 0.049 ^a^	<0.001	0.061	0.798
Tt.Ar (mm^2^)	3.951 ± 0.127 ^a^	3.493 ± 0.104 ^b^	3.517 ± 0.107 ^b^	3.544 ± 0.158 ^a^	3.236 ± 0.137 ^b^	3.284 ± 0.159 ^b^	<0.001	0.119	0.085
Ct.Ar/Tt.Ar (%)	33.1 ± 1.6 ^b^	40.2 ± 1.1 ^a^	41.6 ± 1.1 ^a^	35.0 ± 1.0 ^b^	40.4 ± 0.9 ^a^	41.9 ± 1.2 ^a^	<0.001	0.634	0.384
Ct.Th (mm)	0.110 ± 0.005 ^c^	0.164 ± 0.005 ^b^	0.187 ± 0.004 ^a^	0.120 ± 0.004 ^c^	0.167 ± 0.005 ^b^	0.185 ± 0.003 ^a^	<0.001	0.492	0.139
Ps.Pm (mm)	8.231 ± 0.123 ^a^	7.851 ± 0.104 ^b^	7.939 ± 0.097 ^b^	7.780 ± 0.169 ^†^	7.609 ± 0.159	7.731 ± 0.179	<0.001	0.135	0.046
Ec.Pm (mm)	6.535 ± 0.178 ^a^	5.804 ± 0.153 ^b^	5.724 ± 0.154 ^b^	6.005 ± 0.193 ^a^	5.457 ± 0.184 ^b^	5.440 ± 0.214 ^b^	<0.001	0.134	0.231
Ma.Ar (mm^2^)	2.656 ± 0.139 ^a^	2.097 ± 0.097 ^b^	2.061 ± 0.099 ^b^	2.314 ± 0.131 ^a^	1.937 ± 0.106 ^b^	1.917 ± 0.125 ^b^	<0.001	0.196	0.116
Ecc (no unit)	0.459 ± 0.032 ^a,b^	0.370 ± 0.026 ^b^	0.426 ± 0.021 ^a^	0.459 ± 0.028	0.458 ± 0.034	0.502 ± 0.034	0.041	0.054	0.452

^†^ denotes significantly different (*p <* 0.05) compared to CON within the same month. Different letters in a row denote statistical significance (*p <* 0.05) within a group over time by repeated measures analysis of variance (ANOVA). Data are expressed as mean ± standard error of the mean (SEM), *n =* 8–9/group. BV = bone volume, BV/TV = bone volume fraction, Conn.D = connectivity density, Ct.Ar = cortical bone area, Ct.Ar/Tt.Ar = cortical area fraction, Ct.Th = cortical thickness, DA = degree of anisotropy, Ecc = mean eccentricity, Ec.Pm = endocortical perimeter, Ma.Ar = marrow area, Ps.Pm = periosteal perimeter, Tb.Th = trabecular thickness, Tb.N *=* trabecular number, Tb.Sp = trabecular separation, TMD = tissue mineral density, Tt.Ar = total cross sectional area inside the periosteal envelope, and TV = total volume.

**Table 2:** Ex vivo bone mineral density (BMD), structure and peak load of the femur in 6-month-old female mice whose mothers were exposed to control (CON) or 0.5% hesperidin (HSP) + 0.25% naringin (NAR) diets through pregnancy and lactation.

	CON	HSP + NAR	*p* Value
**Femur BMD (g/cm^2^)**	0.092 ± 0.002	0.090 ± 0.003	0.427
**Femur Neck Trabecular Structure**			
BV/TV (%)	15.6 ± 0.7	15.0 ± 0.9	0.621
Tb.Th (mm)	0.098 ± 0.002	0.099 ± 0.002	0.835
Tb.N (1/mm)	1.591 ± 0.083	1.523 ± 0.102	0.610
Tb.Sp (mm)	0.426 ± 0.014	0.440 ± 0.013	0.462
**Femur Midpoint Cortical Structure**			
Ct.Ar (mm^2^)	1.312 ± 0.034	1.181 ± 0.076	0.101
Ct.Th (mm)	0.264 ± 0.009	0.252 ± 0.010	0.422
**Distal Femur Trabecular Structure**			
BV/TV (%)	11.0 ± 0.9	10.8 ± 2.8	0.932
Tb.Th (mm)	0.081 ± 0.002	0.078 ± 0.005	0.520
Tb.N (1/mm)	1.361 ± 0.105	1.294 ± 0.292	0.813
Tb.Sp (mm)	0.463 ± 0.031	0.544 ± 0.069	0.324
**Femur Neck Peak Load (N)**	23.5 ± 1.2	24.3 ± 1.8	0.697
**Midpoint Peak Load (N)**	25.5 ± 1.1	25.4 ± 2.4	0.602

There were no significant differences in femur outcomes between CON and HSP + NAR groups. Data are expressed as mean ± standard error of the mean (SEM), *n* = 5–9/group. BV/TV = bone volume fraction, Ct.Ar = cortical bone area, Ct.Th = cortical thickness, Tb.Th = trabecular thickness, Tb.N = trabecular number, Tb.Sp = trabecular separation.

**Table 3:** Ex vivo bone mineral density (BMD), structure and peak load of the lumbar vertebra (LV) in 6-month-old female CD-1 mice whose mothers were exposed to control (CON) or 0.5% hesperidin (HSP) + 0.25% naringin (NAR) diets through pregnancy and lactation.

	CON	HSP + NAR	*p* Value
**LV1-3 BMD (g/cm^2^)**	0.075 ± 0.002	0.078 ± 0.004	0.490
**LV2 Trabecular Structure**			
BV/TV (%)	25.1 ± 1.6	22.5 ± 2.3	0.360
Tb.Th (mm)	0.081 ± 0.001	0.087 ± 0.005	0.304
Tb.N (1/mm)	3.106 ± 0.179	2.619 ± 0.228	0.110
Tb.Sp (mm)	0.296 ± 0.012	0.315 ± 0.017	0.359
**LV2 Cortical Structure**			
Ct.Ar (mm^2^)	0.525 ± 0.013	0.523 ± 0.032	0.957
Ct.Th (mm)	0.083 ± 0.002	0.085 ± 0.005	0.706
**LV2 Peak Load (N)**	32.2 ± 2.7	35.8 ± 6.3	0.544

There were no significant differences in bone structure or strength between CON and HSP + NAR groups. Data are expressed as mean ± standard error of the mean (SEM), *n* = 5–9/group. BV/TV = bone volume fraction, Ct.Ar = cortical bone area, Ct.Th = cortical thickness, Tb.Th = trabecular thickness, Tb.N = trabecular number, Tb.Sp = trabecular separation.

## References

[1] Kaludjerovic J., Ward W.E. (2015). Bone-specific gene expression patterns and whole bone tissue of female mice are programmed by early life exposure to soy isoflavones and folic acid. J. Nutr. Biochem..

[2] Kaludjerovic J., Ward W.E. (2010). Neonatal administration of isoflavones attenuates deterioration of bone tissue in female but not male mice. J. Nutr..

[3] Dinsdale E.C., Kaludjerovic J., Ward W.E. (2012). Isoflavone exposure throughout suckling results in improved adult bone health in mice. J. Dev. Orig. Health Dis..

[4] Kaludjerovic J., Ward W.E. (2009). Neonatal exposure to daidzein, genistein, or the combination modulates bone development in female CD-1 mice. J. Nutr..

[5] Chiba H., Kim H., Matsumoto A., Akiyama S., Ishimi Y., Suzuki K., Uehara M. (2014). Hesperidin prevents androgen deficiency-induced bone loss in male mice. Phytother. Res..

[6] Habauzit V., Nielsen I.L., Gil-Izquierdo A., Trzeciakiewicz A., Morand C., Chee W., Barron D., Lebecque P., Davicco M.J., Williamson G. (2009). Increased bioavailability of hesperetin-7-glucoside compared with hesperidin results in more efficient prevention of bone loss in adult ovariectomised rats. Br. J. Nutr..

[7] Horcajada M.N., Habauzit V., Trzeciakiewicz A., Morand C., Gil-Izquierdo A., Mardon J., Lebecque P., Davicco M.J., Chee W.S., Coxam V. (2008). Hesperidin inhibits ovariectomized-induced osteopenia and shows differential effects on bone mass and strength in young and adult intact rats. J. Appl. Physiol. (1985).

[8] Chiba H., Uehara M., Wu J., Wang X., Masuyama R., Suzuki K., Kanazawa K., Ishimi Y. (2003). Hesperidin, a citrus flavonoid, inhibits bone loss and decreases serum and hepatic lipids in ovariectomized mice. J. Nutr..

[9] Habauzit V., Sacco S.M., Gil-Izquierdo A., Trzeciakiewicz A., Morand C., Barron D., Pinaud S., Offord E., Horcajada M.N. (2011). Differential effects of two citrus flavanones on bone quality in senescent male rats in relation to their bioavailability and metabolism. Bone.

[10] Li N., Jiang Y., Wooley P.H., Xu Z., Yang S.Y. (2013). Naringin promotes osteoblast differentiation and effectively reverses ovariectomy-associated osteoporosis. J. Orthop. Sci..

[11] Pang W.Y., Wang X.L., Mok S.K., Lai W.P., Chow H.K., Leung P.C., Yao X.S., Wong M.S. (2010). Naringin improves bone properties in ovariectomized mice and exerts oestrogen-like activities in rat osteoblast-like (UMR-106) cells. Br. J. Pharmacol..

[12] Song B.J., Jouni Z.E., Ferruzzi M.G. (2013). Assessment of phytochemical content in human milk during different stages of lactation. Nutrition.

[13] Zeng X., Bai Y., Peng W., Su W. (2016). Identification of naringin metabolites in human urine and feces. Eur. J. Drug Metab. Pharmacokinet..

[14] Manach C., Morand C., Gil-Izquierdo A., Bouteloup-Demange C., Remesy C. (2003). Bioavailability in humans of the flavanones hesperidin and narirutin after the ingestion of two doses of orange juice. Eur. J. Clin. Nutr..

[15] Rouseff R.L., Martin S.F., Youtsey C.O. (1987). Quantitative survey of narirutin, naringin, hesperidin, and neohesperidin in citrus. J. Agric. Food Chem..

[16] Booth A.N., Jones F.T., Deeds F. (1958). Metabolic and glucosuria studies on naringin and phloridzin. J. Biol. Chem..

[17] Erlund I., Meririnne E., Alfthan G., Aro A. (2001). Plasma kinetics and urinary excretion of the flavanones naringenin and hesperetin in humans after ingestion of orange juice and grapefruit juice. J. Nutr..

[18] Trzeciakiewicz A., Habauzit V., Mercier S., Lebecque P., Davicco M.J., Coxam V., Demigne C., Horcajada M.N. (2010). Hesperetin stimulates differentiation of primary rat osteoblasts involving the bmp signalling pathway. J. Nutr. Biochem..

[19] Trzeciakiewicz A., Habauzit V., Mercier S., Barron D., Urpi-Sarda M., Manach C., Offord E., Horcajada M.N. (2010). Molecular mechanism of hesperetin-7-o-glucuronide, the main circulating metabolite of hesperidin, involved in osteoblast differentiation. J. Agric. Food Chem..

[20] Wu J.B., Fong Y.C., Tsai H.Y., Chen Y.F., Tsuzuki M., Tang C.H. (2008). Naringin-induced bone morphogenetic protein-2 expression via PI3K, Akt, c-Fos/c-Jun and AP-1 pathway in osteoblasts. Eur. J. Pharmacol..

[21] Zhang P., Dai K.R., Yan S.G., Yan W.Q., Zhang C., Chen D.Q., Xu B., Xu Z.W. (2009). Effects of naringin on the proliferation and osteogenic differentiation of human bone mesenchymal stem cell. Eur. J. Pharmacol..

[22] Reeves P.G., Nielsen F.H., Fahey G.C. (1993). AIN-93 purified diets for laboratory rodents: Final report of the American institute of nutrition ad hoc writing committee on the reformulation of the AIN-76A rodent diet. J. Nutr..

[23] Sacco S.M., Saint C., Longo A.B., Wakefield C.B., Salmon P.L., LeBlanc P.J., Ward W.E. (2017). Repeated irradiation from micro-computed tomography scanning at 2, 4 and 6 months of age does not induce damage to tibial bone microstructure in male and female CD-1 mice. BoneKEy Rep..

[24] Otsu N. (1979). A threshold selection method from gray-level histograms. IEEE Trans. Syst. Man. Cyber..

[25] Bruker (2014). Method Note: Bone Mineral Density (BMD) and Tissue Mineral Density (TMD) Calibration and Measurement by Micro-CT Using Bruker-MicroCT CT-Analyser.

[26] Fonseca D., Ward W.E. (2004). Daidzein together with high calcium preserve bone mass and biomechanical strength at multiple sites in ovariectomized mice. Bone.

[27] Breinholt V., Larsen J.C. (1998). Detection of weak estrogenic flavonoids using a recombinant yeast strain and a modified MCF7 cell proliferation assay. Chem. Res. Toxicol..

[28] Kuiper G.G., Lemmen J.G., Carlsson B., Corton J.C., Safe S.H., van der Saag P.T., van der Burg B., Gustafsson J.A. (1998). Interaction of estrogenic chemicals and phytoestrogens with estrogen receptor beta. Endocrinology.

